# Measuring Digital PCR Quality: Performance Parameters and Their Optimization

**DOI:** 10.1371/journal.pone.0153317

**Published:** 2016-05-05

**Authors:** A. Lievens, S. Jacchia, D. Kagkli, C. Savini, M. Querci

**Affiliations:** Molecular Biology and Genomics Unit, European Commission - Joint Research Centre, Institute for Health and Consumer Protection, 21027 Ispra (VA), Italy; Hospital Authority, CHINA

## Abstract

Digital PCR is rapidly being adopted in the field of DNA-based food analysis. The direct, absolute quantification it offers makes it an attractive technology for routine analysis of food and feed samples for their composition, possible GMO content, and compliance with labelling requirements. However, assessing the performance of dPCR assays is not yet well established. This article introduces three straightforward parameters based on statistical principles that allow users to evaluate if their assays are robust. In addition, we present post-run evaluation criteria to check if quantification was accurate. Finally, we evaluate the usefulness of Poisson confidence intervals and present an alternative strategy to better capture the variability in the analytical chain.

## Introduction

About two decades ago, digital PCR was developed as a potential alternative quantification strategy [1, 2] and in the wake of technological advances in the field of nanofluidics, several digital PCR platforms have recently entered the market. Digital PCR is based on the concept of limiting dilutions. Practically a reaction is split into a large number of (nanoliter) sub-reactions so that individual target copies are separated by the process of partitioning. After thermal cycling and read-out this leads to the classification of each partition as either positive (containing target) or negative (no target present).

The distribution of the target molecules across the partitions can be seen as a Poisson process (the targets end up in partitions independently and with a fixed rate). Poisson statistics thus allow the calculation of the initial number of targets from the number of positive and negative partitions. As a consequence, digital PCR is an absolute quantification strategy by default. This is very different from qPCR, which is based on the proportionality between fluorescence & DNA mass and where quantification is always relative. Therefore, calibration curves are needed to invoke absolute quantification in a qPCR setting.

A recent survey among European food analysis laboratories indicated that a growing proportion of them are investing in digital PCR platforms and that this technique is increasingly applied in a routine setting (*e.g.* GMO analysis, food fraud, species identification). This ongoing permeation of digital PCR into a legislative setting signals the need for application guidelines and for a harmonization effort. Here, we have established several criteria to measure the performance of digital PCR assays and have set the limits for the corresponding parameters. Further, we have explored a range of theoretical and practical aspects of dPCR in order to gauge which practices may help establish a robust and reliable framework for digital quantification of the DNA targets in food and feed samples.

## Material and Methods

### DNA Samples and PCR reactions

All DNA extracts were quantified fluorometrically using Picogreen (Molecular Probes) and a fluorometer (Biorad Versafluor). The amount of template copies was calculated from the DNA quantities using haploid genome weights [3].

**Samples** were prepared from dry materials (CRM and seed material). DNA was extracted using a CTAB based method adopted from [4]. The exact protocol for DNA extraction is available in S1 File.

**real time PCR** reactions were performed in 25*μ*l using primers from [5–13] (see S1 Table). Probe-based reactions were run using Taqman Universal Mastermix (Life Technologies), primers and probes were ordered from Eurogentec. All reactions were amplified in ABI microamp 96-well plates using either an Applied Biosystems ABI7900 or ABI7500 (Life Technologies). A single thermal cycling protocol was used for all real time PCR reactions: 10min 95°C, 60× (15sec 95°C, 1min 60°C). Results were analysed & exported using the SDS 2.4.1 software.

**digital PCR** reactions were performed using the Biorad QX200 digital droplet platform using Twin.Tec 96 well PCR plates (Eppendorf). Initial volume of the reaction mixture was 20*μ*l which, together with the droplet generating oil, resulted in a final PCR volume of approx. 45*μ*l. Reactions were set up using Probe Supermix (Biorad), primers and probes were ordered from Eurogentec. Thermal cycling was performed on a Biorad C1000 using the following thermal cycling protocol: 10min 95°C, 45× (15sec 95°C, 1min 60°C), 10min 98°C. Results were analysed & exported using the Quantasoft 1.6.6.320 software.

**digital touchdown PCR** was performed as described in [14]. The final thermal cycling protocol used was: 10min 95°C, 30× (15sec 95°C, 1min 63°C), 15× (15sec 95°C, 1min 60°C), 10min 98°C.

**Temperature gradient** was set up using the built-in function of the C1000 thermal cycler. Each row runs at a different temperature. Per column eight technical repeats of each method were loaded. The gradient protocol was: 10min 95°C, 45× (15sec 95°C, 1min 62-56°C), 10min 98°C. The individual row temperatures were: 62, 61.6, 60.9, 59.8, 58.4, 57.3, 56.5, and 56°C (from row A to H).

**Cycling gradient** was carried out by cutting a dPCR plate in four strips. Each strip was loaded with the same reactions (i.e. one vial of reaction mix for all strips). The entire plate was loaded into the thermal cycler (programmed for 90 cycles). Strips were removed from the thermal cycler after 45, 60, 75, and 90 cycles (by pausing the run during the denaturation stage).

**Sonication** was carried out using a Sonics Vibra-cell VCX750 at 30% amplitude. Samples were prepared at 12.5 ng/*μ*l in a 150 *μ*l volume and sonicated for either 3, 6, 9, 12, or 15 seconds.

**Repeated reactions** for inspecting the confidence interval (CI) coverage were created from independently diluted samples. A stock solution of 2% w/w roundup ready soybean (GTS-40-3-2) at a concentration of 100 ng/*μ*l was used to prepare 48 independent dilutions (final concentration 10 ng/*μ*l, single dilution step). Each dilution was analysed in 4 replicates using duplex digital droplet PCR (ddPCR) reactions (targets: Lectine, event GTS 40-3-2) with a total of 30 ng of template DNA per reaction (respectively, ≈26000 and ≈520 target molecules per reaction). The stock solution further served to make 48 reaction mixes (DNA + mastermix, primers, etc.), which were analysed in duplicate (a total of 300 ng of template DNA per reaction, ≈260000 Lectine and ≈5200 event GTS 40-3-2 target molecules per reaction). All repeats (single dilution) were run on the same droplet generating strip.

**PCR enhancers** used were: DMSO (Sigma-Aldrich) used at 2% and 5% final concentration, D-(+)-Trehalose dihydrate (Sigma-Aldrich) used at 0.2M final, and Tween 20 (Sigma-Aldrich) used at 0.5% final. All enhancers were prepared in a 10× concentration and then added to the reaction mix.

### Statistical Models

We mainly follow the statistical models for digital PCR as presented in [15, 16]: let *n*_*t*_ be the total number of partitions for which we have a read-out and *n*_âŠ–_ the number of negative droplets, we then estimate let *p*_0_ (chance of an empty droplet) and *λ* (the average number of targets per droplet) as:

$${\hat{p}}_{0}\;=\;\frac{n_{⊖}}{n_{t}}$$ (1)

$$\hat{\lambda }\;=\;-\text{ln}\left({\hat{p}}_{0}\right)$$ (2)

The ratio of GMO in the sample (in haploid genome equivalents) is then given by

$$\begin{array}{c} {\hat{r}}_{GM}=\frac{{\hat{\lambda }}_{tr}}{{\hat{\lambda }}_{en}} \end{array}$$ (3)

where ${\hat{\lambda }}_{tr}$ and ${\hat{\lambda }}_{en}$ are the concentration estimates for the transgene and endogene respectively. Note that calculating the GM ratio does not require the droplet volume to be known, it is however assumed to be constant. The 95% Confidence Bounds (CB) can be calculated for each of the individual *λ* estimates:

$$\begin{array}{c} {\hat{\lambda }}_{CB}=\hat{\lambda }\pm 1.96\cdot \sqrt{\frac{n_{t}-n_{⊖}}{n_{t}\cdot n_{⊖}}} \end{array}$$ (4)

Whereas the confidence bound for the ratio of transgene to endogene is obtained using Fiellers theorem [17]:

$$\begin{array}{c} {\hat{r}}_{GM,CB}=\frac{{\hat{\lambda }}_{tr}\cdot {\hat{\lambda }}_{en}\sqrt{{\hat{\lambda }}_{tr}^{2}\cdot {\hat{\lambda }}_{rn}^{2}\left(E^{2}-{\hat{\lambda }}_{en}^{2}\right)\left(T^{2}-{\hat{\lambda }}_{tr}^{2}\right)}}{{\hat{\lambda }}_{tr}^{2}-T^{2}} \end{array}$$ (5)

where $E=|{\hat{\lambda }}_{en}-\lambda_{en,CB}|$ and $T=|{\hat{\lambda }}_{tr}-\lambda_{tr,CB}|$ with CB being the relevant confidence bound (upper or lower).

For additional details we refer the reader to the original publications [15, 16].

### Digital PCR droplet categorization

For the categorization of the compartments’ fluorescence readings into positive (containing target DNA, high fluorescence), negative (no target DNA, low fluorescence), and rain (intermediate fluorescence) we used a procedure based on kernel density estimation. Hereunder we will briefly explain the basics of the process; the algorithm is available as an R-script for further inspection (see S2 and S3 Files).

The algorithm takes the compartments’ (final) fluorescence readings as the only input. In a first step, the Gaussian kernel density of the fluorescence is estimated using the density function with a minimum bandwidth of 50. Subsequently the most prominent density peaks are identified using a sliding window approach. The details for the subsequent steps differ depending on the number of peaks that were found (*i.e.* one, two, or three or more) but in general, the total fluorescence bandwidth is split into parts and in the outermost parts the fluorescence position of the density peak is taken as the initial estimate of the population median (either positives or negatives depending on which part is inspected). Assuming normality, the standard deviation of the population distributions is then estimated as half the peak width at 60,65% of its maximum height.

Next, the initial estimates are refined using an iterative process: the values in a range of $a\cdot \hat{\sigma }$ (initially, *a* = 4) around the median are used to recalculate the median and median average deviation as robust estimators for the population parameters. In addition, as deviations from normality were found to be common, the kurtosis of the population is inspected. The higher the kurtosis, the higher *a* needs to be to cover 99% of the values. Let *k* be the kurtosis of the distribution, then *a* is updated using the following equation:

$$\begin{array}{c} a=4.55+0.35\cdot \text{log}\left(k\right)+0.045\cdot \text{log}{\left(k\right)}^{2} \end{array}$$ (6)

This equation is based on the analysis of in house data. For a large number of dPCR fluorescence ‘populations’ the kurtosis was regressed against the number of *σ* needed to reach 99% coverage.

Three iterations of the above process were found to be enough to reach stability in the first decimal place. Readings falling within the $\hat{\mu }\pm a\cdot \hat{\sigma }$ range are categorized as belonging to either the positive (*p*) or negative (*n*) population. The limits of this range are regarded as the respective peak bases for calculating the resolution. Readings between ${\hat{\mu }}_{n}+a_{n}\cdot {\hat{\sigma }}_{n}$ and ${\hat{\mu }}_{p}-a_{p}\cdot \hat{\sigma_{p}}$ are classified as rain.

For the final quantification a threshold *θ* is placed *above* the uppermost limit of the negative population to minimize the probability of misclassifying negative droplets. Droplets with fluorescence readings larger than *θ* are counted positive, the ones smaller than *θ* are counted negative. For our data set, we found $1.5\cdot a_{n}\cdot {\hat{\sigma }}_{n}$ to be a sufficient distance from the median to minimize negative misclassification. The threshold value is thus calculated as:

$$\begin{array}{c} \theta ={\hat{\mu }}_{n}+1.5\cdot a_{n}\cdot {\hat{\sigma }}_{n} \end{array}$$ (7)

The algorithm further performs several checks to make sure that population boundaries do not overlap and that the threshold is placed correctly relative to the populations. In case of problems, the threshold and/or the boundaries in questions are replaced with estimates that are not based on population parameters. Instead, these new estimates are based on the minima of the kernel density as found by the sliding window approach mentioned above.

### Data processing

All calculations and curve fitting were done using R version 2.15.3 [18]. The data were exported from the droplet reader and imported into R. Parameter modelling was accomplished using the standard linear modelling function (lm) in combination with non-linear curve fitting using the Levenberg-Marquardt algorithm [19, 20] available through the package ‘minpack.lm’ version 1.1-5.

R scripts of all simulations and parametric bootstraps mentioned throughout the text are available in S4 File. In addition, there is a github page (http://www.github.com/Gromgorgel/ddPCR) that contains usage instructions, R-code examples, as well as the entire data set underlying the findings this publication.

## Results and Discussion

### Part I: method performance criteria

Many digital PCR performance requirements are identical to qPCR (applicability, practicability, specificity, dynamic range, trueness) and will not be considered here. Instead, we focus on the aspects of digital PCR that are fundamentally different from real time PCR: *i.e.* compartmentalization and the subsequent classification into positive and negative compartments.

If a PCR assay is specific and efficient, the main source of error in digital quantification is caused by the misclassification of droplets. Thus, the main goal of the criteria should be to ensure a robust separation of positives and negatives allowing accurate classification of compartments and thus reliable quantification. Three criteria that allow to meet this requirement are (**I**) single amplification product (there should only be two fluorescence populations) (**II**) peak resolution (as a measure of the separation between positives and negatives), and (**III**) the amount of stragglers or ‘rain’ (*i.e.* droplets that have an intermediate fluorescence and do not seem to belong to either the positive or negative population). Several other criteria were considered (gain, bandwidth, and signal to noise ratio) but were either found to be not very informative or overlapping with the former (see S5 File for definitions).

In the following sections, we will properly define these criteria and set their limits, taking into account the existing performance requirements for analytical methods of GMO testing [21, 22] which are widely used among food testing laboratories to benchmark their real time PCR methods. Specifically, the requirement that the relative error should be below or equal to 25% is used as a starting point for the calculation of parameter limits. We propose the condition for the measurement of the dPCR performance criteria to be *λ* = 0.7. This corresponds to a situation in which approximately half of the compartments are positive and thus allows to gauge both the rain and the dispersion of the droplets without being biased by the fact that one population is larger than the other.

We have evaluated the usefulness of these criteria and their limits by applying them to 12 validated qPCR assays that were transferred to digital PCR. S1 Table lists all primers and probes sequences. These methods cover fours species and eight GM events. The results of these experiments are summarized in S2 and S3 Tables of and are presented in more detail throughout the remainder of this document.

### Single amplification product

**Fig 1:**
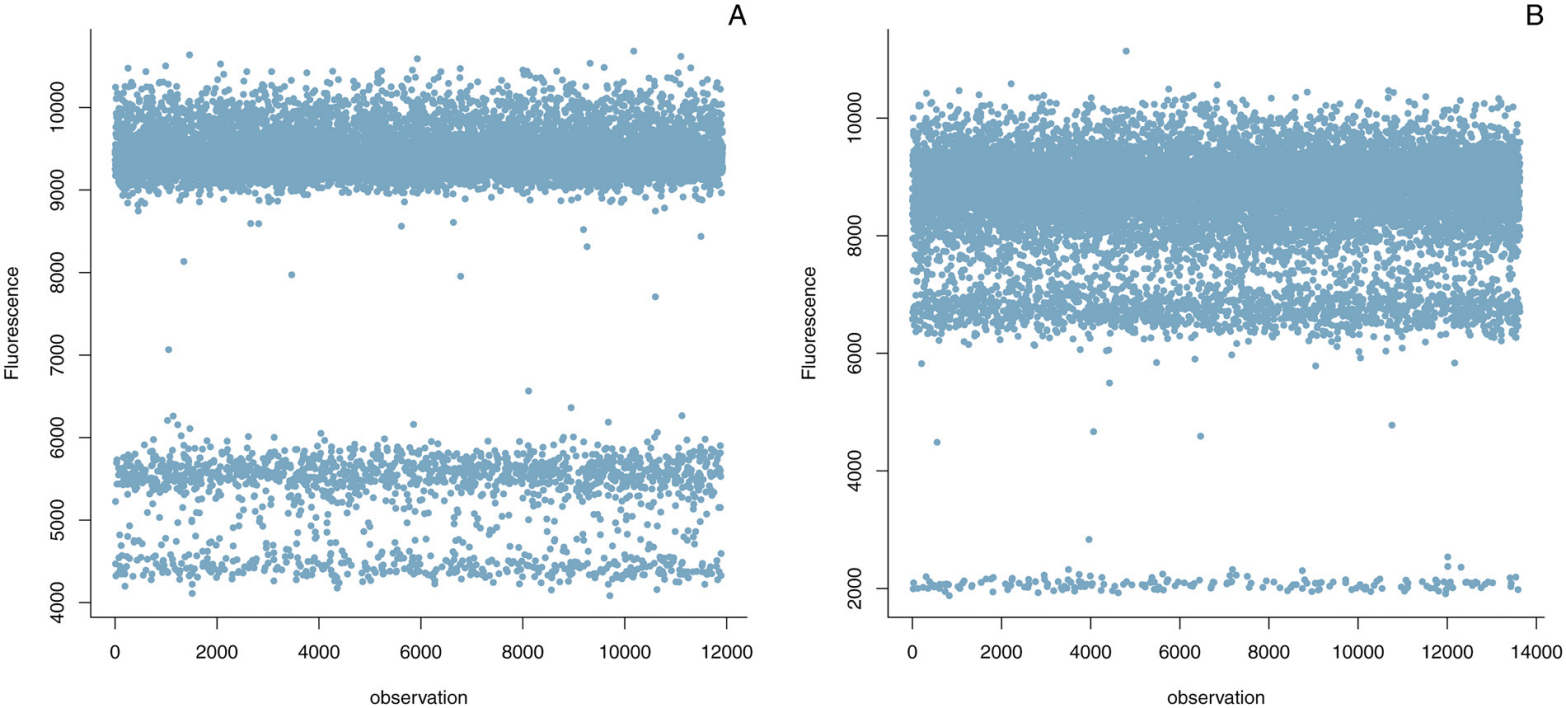
Illustration of multiple fluorescence populations. **Panel A** shows the results for the *acp1* target, an additional population of fluorescence measurements is visible with values closely situated to the negatives. **Panel B** shows the results for the *cruA* target, an additional population of fluorescence measurements is visible with values closely situated to the positives. Contrary to ‘rain’, the measurements with intermediate fluorescence are not uniformly spread.

In a standard real time PCR reaction, the fluorescence measured is the sum of all amplification processes in the reaction mixture. It is therefore not possible to distinguish between different amplification products in the same reaction (*e.g.* the amplification of closely related sequences). In digital PCR, the compartmentalisation allows such a distinction. This property can be exploited to yield dye-based multiplexing [23] and can uncover specificity issues in probe-based reactions. In real time PCR, unintended amplification products (*i.e.* non-perfect match) usually amplify at a lower efficiency. This results in an endpoint fluorescence that is lower than for the actual target (after a standard run of 45 cycles). As a consequence, these amplifications show up in digital PCR as an additional population of droplets with fluorescence values between the true positive and negatives (see Fig 1).

It is possible that these unintended products do not significantly accumulate during real time PCR due to the competition for reagents. In addition, the formation of additional reaction products does not necessarily represent a total loss of specificity: *i.e.* the amplicons may result from related sequences present only in the analyte of interest (*e.g.* a specific species or variety). In the latter case, specificity at the real time PCR level is not affected.

However, the presence of multiple populations of droplets may complicate the digital analysis, affect the separation of positives from negatives, and ultimately lead to misclassification of droplets. We therefore require dPCR assays to only form 1 amplification product (whose template is preferentially present in one copy per haploid genome for optimal quantification). Out of the twelve qPCR assays tested, two showed multiple bands when transferred to digital PCR (*i.e.* the ones targeting *acp1* and *cruA*, see Fig 1).

#### Optimizing for a single amplification product

If more than two populations of droplets are present, one usually amplifies at maximal efficiency (positives) whereas the intermediate population accumulates fluorescence at a lower rate. By raising the annealing temperature and thus increasing the specificity of the primer binding, one can usually increase the difference in efficiency between the intended target and co-amplified target, often to the point where the co-amplified population merges with the population of negative droplets.

**Fig 2:**
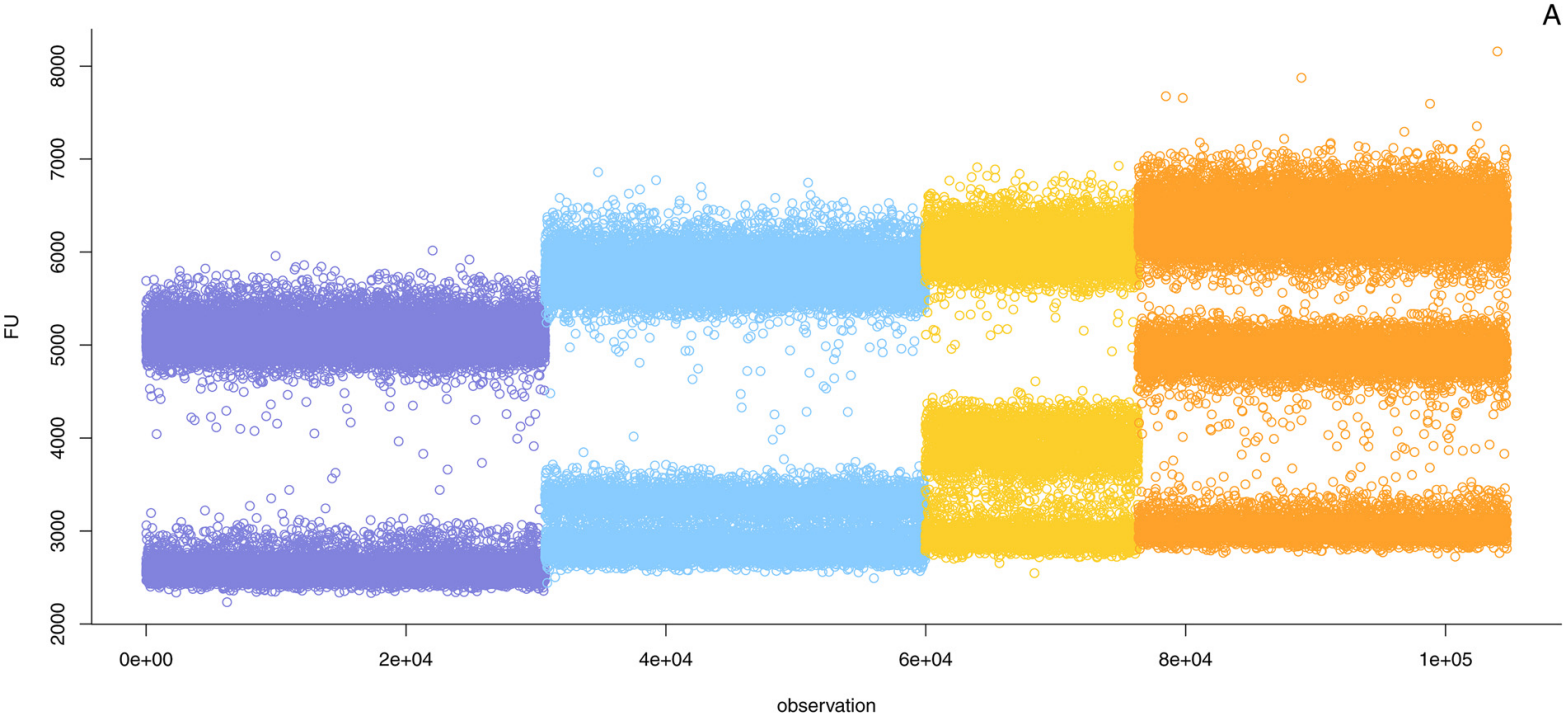
Running a gradient to reduce the effect of co-amplification in digital PCR. The figure shows the observed droplets for 4 temperatures: 62, 59.8, 58.4, and 56°C (from left to right). All reactions were run at *λ* ≈ 1. As the annealing temperature is raised the specificity of the reaction increases and efficiency of the co-amplification is reduced, until the undesired droplet population merges with the negative population.

Running a temperature gradient of an assay, one can select the conditions that allow for optimal distinction between positive and negative droplets (see Fig 2). However, changing the annealing temperature may also influence the efficiency of the amplification of the intended target. This can result in a decrease in peak separation between positives and negatives, which affects the performance of the assay. Digital touchdown PCR [14] can be used to counter the decrease in separation. In such protocol, 30 cycles at an increased temperature are followed by 15 cycles at a standard temperature. This strategy allowed us to obtain a single amplification product for *acp1* at an acceptable separation. However, it did not work for *cruA*, the difference in efficiency between the intended and the secondary target does not seem to be large enough to be exploited this way. In such cases it may be more straightforward to design new primers for the target sequence.

### Resolution

Peak resolution is a concept from the field of HPLC (High Pressure Liquid Chromatography) that translates well to digital PCR when applied to the density plots of the droplet fluorescence readings. The resolution of a digital assay (*R*_*s*_) is a quantitative measure of how well the two populations (positive and negative) can be differentiated in a linear separation. It is defined as the difference in fluorescence between the two peaks, divided by the combined widths of the peaks:

$$\begin{array}{c} R_{s}=\frac{2\cdot (t_{p}-t_{n})}{w_{p}+w_{n}} \end{array}$$ (8)

**Fig 3:**
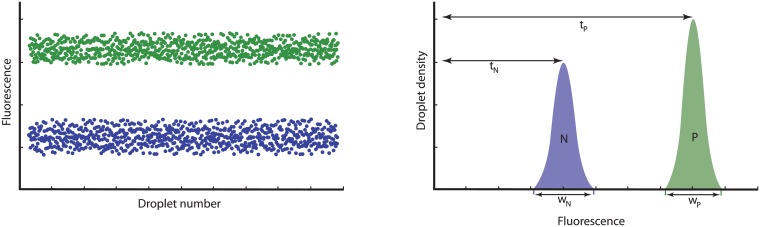
Illustration of the concept of resolution. The left-hand figures show the droplet readout, the right-hand figures show the corresponding density plots. *t*_*n*_ and *t*_*p*_ show the fluorescence positions with the highest density in the negative and positive droplet clouds respectively. *w*_*n*_ and *w*_*p*_ represent the width of the density peaks at their base.

Where the subscript *p* indicates the population with the higher fluorescence (positive, as opposed to *n* for the negatives). The variables *t* and *w* are the peak fluorescence and peak width respectively (see Fig 3 for an illustration).

Essentially, the resolution corresponds to how well the fluorescence of the droplet populations is separated. Hence, a higher resolution represents a lower chance of misclassifying droplets. Although a resolution of 2 represents a complete separation, we propose *R*_*s*_ = 2.5 as a minimum to allow for a certain amount of deterioration of the resolution in more difficult samples (*e.g.* inhibited or degraded). For none of the PCR targets we inspected this criterion presented a problem, with *R*_*s*_ values ranging between 2.7 and 6.6 (after optimization, see below).

All resolution values in this publication were calculated directly from the dPCR fluorescence measurements using the R algorithm described in the ‘materials and methods’ section and available in S3 File, but can be manually estimated from the density plots in the digital PCR software. However, since not all software may provide the user with density plots or the ability to edit/export fluorescence values, we also propose an alternative (manual) strategy in S5 File. The latter allows to calculate the resolution based on the relative width of the ‘bands’ visible in the droplet plots of most dPCR software.

#### Optimizing the resolution of a method

The resolution of a PCR method is not a fixed quality, but is influenced by a number of reaction conditions and can thus be optimized to a certain degree. In terms of real time PCR, the negative droplets represent fluorescence measurements for the baseline (base fluorescence value), whereas the positives represent endpoint fluorescence measurements (plateau fluorescence value). Both are predominantly determined by (I) quenching efficiency, (II) single amplicon fluorescence, and (III) primer-probe concentration. The latter being the most straightforward to adjust, whereas the former involve experimenting with different fluorophores and quenchers.

**Fig 4:**
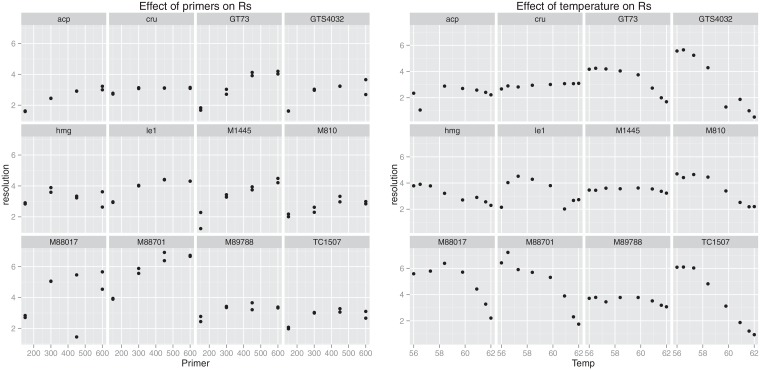
Effect of reaction conditions on digital PCR resolution. **Panel A** shows the effect of primer concentration. For each of the twelve targets, four primer concentrations were tested (150, 300, 450, and 600 nM). Probe concentrations are such that the primer to probe ratio is the same as for the validated conditions (see S1 Table). **Panel B** shows the effect of annealing/elongation temperature. For each of the twelve targets, eight temperatures between 62 and 56°C were tested.

By increasing the primer and probe concentrations in a reaction, one increases the maximum number of copies that can be produced in that reaction and thus the height of the plateau. However, there is only a significant increase in plateau to the point where the primers are no longer the limiting factor [24]. Another effect of adding more primer and probe is the increase in base fluorescence, which is detrimental to the resolution. As a result, the resolution as a function of primer/probe concentration usually has a maximum or plateau beyond which further addition of primers is useless (see Fig 4A). For nearly all methods tested, this point lay in the vicinity of 500 nM of primers (while keeping the primer:probe ratio the same as the validated qPCR conditions). Table 1 lists the primer conditions selected for each target along with the corresponding performance parameters.

**Table 1:** Performance parameters for each target under optimised conditions.

target	[Primer]	[Probe]	*R*_*s*_	% Rain	#pop
*Acp1*^†^	450	150	3,09 ± 0,05	0,009 ± 0,0006	2
*CruA*	200	200	2,87 ± 0,02	0,122 ± 0,0037	3
*le1*	450	150	4,13 ± 0,02	0,009 ± 0,0007	2
*hmg*	300	180	3,60 ± 0,19	0,019 ± 0,0014	2
MON810	450	270	3,98 ± 0,22	0,008 ± 0,0004	2
TC1507^‡^	300	150	2,92 ± 0,20	0,014 ± 0,0020	2
MON88017^‡^	450	150	6,35 ± 0,64	0,003 ± 0,0004	2
MON1445	450	150	3,89 ± 0,15	0,004 ± 0,0007	2
GTS-40-3-2	300	100	2,70 ± 0,07	0,014 ± 0,0022	2
MON88701	450	190	6,60 ± 0,30	0,011 ± 0,0005	2
MON89788	300	100	3,71 ± 0,17	0,014 ± 0,0032	2
GT73	450	150	4,27 ± 0,12	0,014 ± 0,0015	2

[Primer] and [Probe] give the final concentration of primers and probe in the reaction mix (nM), *R*_*s*_ gives the mean peak resolution and its standard deviation (calculated over four repeats). %Rain gives the mean ratio partitions that were categorized as rain to of the total number of partitions per reaction and its standard deviation (calculated over four repeats). #pop gives the number of fluorescence populations detected by our algorithm. Targets marked with † made use of digital touchdown PCR, targets marked with ‡ had their template DNA sonicated for three seconds. Note that for *CruA* the ‘third’ or intermediate population was entirely categorized as rain, explaining the high value in that field.

Another option for optimizing the resolution is the adjustment of the annealing and/or elongation temperature. Changing the annealing temperature to lower values may yield an increase in digital resolution (see Fig 4B). The size of the increase is largely method dependant, with some methods barely showing a change in resolution (*e.g.* the cruciferin and MON1445 targets) whereas others are affected dramatically (*e.g.* TC1507 and GTS-40-3-2). However, lowering the annealing temperature affects the specificity of the method and additional controls should be performed to confirm that the new reaction conditions do not result in unspecific amplification.

### Percentage of ‘rain’

In many dPCR reactions there are compartments that seemingly fail to belong to either the positive or negative population. These compartments have an intermediate fluorescence level and are colloquially referred to as ‘rain’ or ‘drag’. In this paper we have set fluorescence limits on the positive and negative populations in terms of a number of standard deviations around their median (based on their kurtosis, see materials and methods). We therefore define the ‘rain’ as the compartments whose fluorescence readings are between the maximal negative fluorescence and the minimal positive fluorescence. With a strict definition of rain, we can inspect its properties and measure its behaviour in response to changes in experimental conditions.

Contrary to the distribution of intermediate fluorescence readings in case of multiple amplification products, rain does not seem to have a pronounced distribution: it is often equally spread over the ‘empty’ space between positives and negatives and is close to uniformly distributed (data not shown). In order to be able to set a meaningful threshold for the amount of rain that we can tolerate, we need to understand the nature these compartments: *i.e.* are they essentially positive (containing the target sequence), negative (void of target), or mixed (random).

We have set up a series of straightforward experiments to determine the nature of the rain for two targets that were observed to produce the most rain (*i.e.* TC1507 and MON88017). The first experiment consisted of running a serial dilution series for both targets and inspecting the number of compartments categorized as rain. Specifically, three scenarios were investigated: **(I)** the number of rain compartments correlates with the number of negative compartments, indicating the rain does not contain target and should be categorized as negative; **(II)** correlation with the amount of positives, indicating the rain contains the target sequence and should be categorized as positive; **(III)** correlation with the total number of compartments, indicating a more random process and complicating the threshold placement.

**Fig 5:**
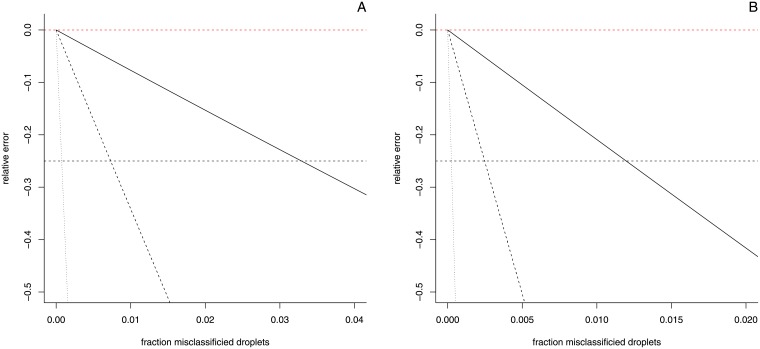
Effect of additional cycles and sonication on the amount of rain. Round dots represent the results for TC1507. Triangles represent the results for MON88017. **Panel A** shows the effects of running additional cycles: four conditions of increasing number of cycles were tested (45, 60, 75, and 90 cycles). **Panel B** shows the effects of sonication: six conditions of increasing sonication were tested (ranging from 0 seconds to 15 seconds in steps of 3 seconds).

For both targets the second possibility was found to be the case (see S1 Fig). This suggests that the rain compartments do contain the target sequence, but that they are unable to amplify at the same efficiency as the main target population. The second experiment further confirmed that, at least for these two targets, this indeed was the case: by running the same reactions for an increasing number of cycles we were able to decrease the amount of rain by allowing these inefficient reactions to accumulate more amplicon copies (see Fig 5A and S2 Fig).

**Fig 6:**
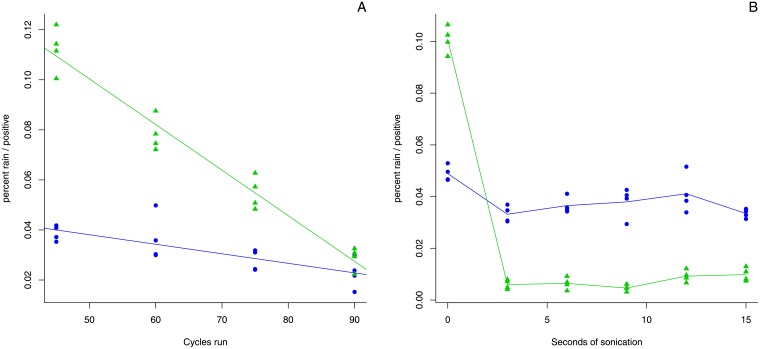
Error as a result of droplet misclassification (positives misclassified as negative). The effect of misclassification is shown for quantification at three levels (1%, 0.5%, and 0.1%) indicated by different line styles (solid, dashed, and dotted respectively). The black horizontal dashed line represents the amount of misclassification that can be tolerated before 25% error is reached. **Panel A** shows the results for *λ* = 3. **Panel B** shows the results for *λ* = 1. For the 5% level, 3.3% of the positive droplets can be misclassified before 25% error is reached (1.2% at *λ* = 1). For the 1% level, this is only 0.73% (0.25%) and for the 0.1% level only 0.075% (0.025%).

In order to set a limit to the amount of rain that can be allowed in digital PCR reactions we consider the misclassification of compartments. Specifically, the classification of positives as negative since the rain in our reactions most likely contains the target sequence. At low values of *λ* such misclassification leads to a significant underestimation of the number of positives, whereas at high values of lambda the number of negatives is significantly overestimated. S3 Fig illustrates the error resulting from both cases as well as the transition between these two extremes. For GMO quantification (in which the transgene to endogene ratio is determined), we can simulate the effect of the misclassification of transgene compartments on a theoretical base and inspect the resulting relative error on the final quantification result (we assume the endogene to be quantified correctly). The results of these simulations for different values of *λ* and for different percentages of target sequence are presented in Fig 6.

For a *λ* value of 3 and a transgene level of 5%, about 3.3% of the positives can be misclassified before 25% error is reached (1.2% at *λ* = 1), while at the 1% and 0.1% level, this is only 0.73% (0.25%) and 0.075% (0.025%) respectively. Assuming 20 000 droplets were generated this translates into misclassifying 658 (239), 146 (50), and 15 (5) droplets for the 5%, 1% and 0.1% levels respectively. These results underline the importance of categorizing the rain as positive and of working at a sufficiently high value of *λ* to reduce the impact of misclassification.

However, the fact that even after 90 cycles of amplification there is still a significant amount of rain combined with the fact that these rain readings are more or less uniformly distributed between the baseline and the plateau (rather than clustered near the positive cloud) seems to indicate that after a standard reaction (40 or 45 cycles) the cloud of negatives may still house a population of rain compartments that have not yet had the time to accumulate fluorescence above the baseline level. As a consequence, these compartments would always be misclassified, underscoring the importance of relatively rain-free methods.

Thus, the main question relating to how much rain we can tolerate becomes: how many rain partitions are ‘hiding’ in the negative cloud (and are thus always misclassified). Consider the 1% quantification level with endogenous *λ* = 1 and assume a worst case scenario in which 10% of the rain is concealed by the negative cloud. Since setting the threshold just above the negative cloud will correctly classify 90% of the compartments, we can theoretically tolerate 10 times the number of compartments needed to reach 25% error. Which, in this case, is 50 × 10 = 500, being approximately 2.5% of the total number of compartments generated (20000). Hence, we propose the latter as a rule of thumb for the maximum amount of rain in a given reaction.

#### Avoiding and removing Rain

In our hands, rain seems to be largely method dependent. This indicates that it may primarily be a property of either the primers or of the target sequence. Therefore, primer design may be the key element in avoiding rainy reactions. However, re-designing primers may not be an option for all targets. For instance, GMO event-specific methods are designed to cover the junction between transgenic insert and host genome, leaving little room for improvement. In such cases, any of the following options may be explored:

**Running more cycles** was shown to reduce rain as the ‘delayed’ or inhibited targets are allowed to amplify further and more partitions migrate to the positive band. This is the least labour intensive, yet the most time consuming option as the amount of rain decreases only linear with the amount of cycles run (see Fig 5A).

**Sonication** was shown to reduce the amount of rain in both reactions tested. In one case (MON88017) three seconds of sonication were sufficient to remove almost all rain (see Fig 5B and S4 Fig). These results provide an indication that in some cases rain may be caused by secondary structures in the target DNA. However, the absence of such a spectacular result in the other case (TC1507) seems to indicate that this is only one of several mechanisms that may lead to the formation of rain droplets.

**PCR enhancers** can be added to the reaction to increase the efficiency of amplification in the rain droplets. Of all the enhancers that were tested in this study, few were found to be compatible with the Biorad dPCR chemistry (*i.e.* DMSO and trehalose), and the results were not very promising (see S5 Fig). However, several other enhancers have been described in the literature (*e.g.* dithiotreitol, Beatine monohydrate, NP-40, formamide) and may be more effective in removing rain.

**Annealing conditions** may be changed in an attempt to yield a more efficient amplification process. However, in this study lowering the annealing/elongation temperature did not result in a significant reduction in the amount of rain compared to the standard conditions (60°C), but higher annealing/elongation temperatures did increase the amount of rain.

### Part II: Post-Run evaluation

One of the key tasks an operator has to perform in the post-run analysis is to evaluate the quality of the data produced by the reaction. For real time PCR, this means taking into account positive-, negative-, and no-template controls, evaluation of the melting curve (in dye based chemistry), evaluation of the amplification profile, etc. In most digital systems, there is only endpoint read-out of the partitions which provides less information as to how the amplification process performed.

We propose three reaction aspects that can be inspected before deciding to accept or discard it for further analysis: **(I)** the concentration of the target (is it sufficient to allow accurate quantification?), **(II)** the degree of compartmentalization (has a sufficient number of partitions been generated to allow accurate quantification?), and **(III)** the use of repeats to gauge variability in the results (rather than relying on Poisson confidence intervals).

### Target concentration and dPCR quantification

**Fig 7:**
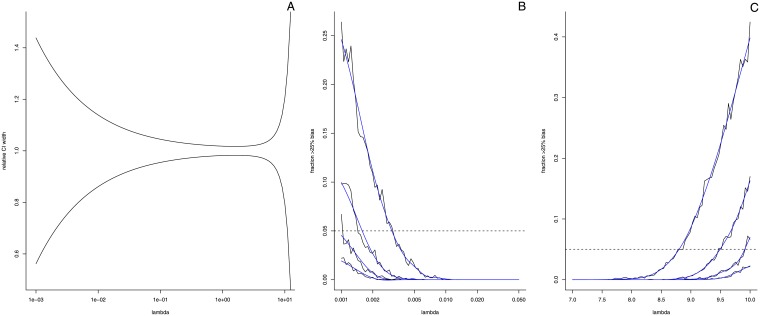
Digital PCR confidence limits. **Panel A** shows the relative width of Poisson confidence intervals as a function of the number of target sequences per partition (*λ*). **Panel B** and **C** illustrate how many percent of the *λ* estimates of a 20 000 partition system contain 25% or more error as calculated via parametric bootstrap for different numbers of repeated analysis (single reaction, duplicate, triplicate, and four repeats). Replicates are averaged to obtain the final estimate. The black lines show the actual percentages obtained, the blue lines show a smoothed version of the curve. The dashed horizontal line represents five percent.

As mentioned in several other papers, there is an optimal concentration of target molecules per partition (*λ*) that should yield the least random sampling variability (and thus the narrowest confidence bounds). By plotting the confidence bounds obtained using Eq 3 we can inspect the effect of the target concentration on the width of the confidence interval (see Fig 7). From these curves we infer that the optimal value of *λ* for absolute quantification is ≈1.6, *i.e.* if random sampling variation as described by the Poisson process is the only source of variability. For a partition size of 0.85nl this translates to an optimum of ≈1870 target copies per *μ*l or ≈37400 per 20 *μ*l. For a partition size of 6nl this translates to ≈265 per *μ*l or ≈2650 per 10 *μ*l for optimal conditions.

In order to find an upper and lower bound for optimal quantification we can inspect the fraction of reactions that have more than 25% error due to Poisson variation. Using a bootstrap approach we can find the values of *λ* for which this fraction reaches 5% (*i.e.* 95% of all reactions at this value of *λ* result in quantification with less than 25% error). We performed 2000 bootstraps each for 75 logarithmically spaced values of *λ* at both the high and low end of the dynamic range of two digital PCR systems (20 000 and 765 partitions). In each bootstrap, partitions were generated from a Poisson distribution with the given *λ* and were subsequently used to re-estimate *λ*. The latter value was then compared to the input to inspect how many bootstraps yielded more than 25% error. Table 2 shows the results for the bootstrap analysis (also see Fig 7B and 7C). In addition, the table also shows how these limits shift when instead of a single reaction, multiple repeats are used (*i.e.* averaged) to estimate *λ*.

**Table 2:** Results from the bootstrap analysis.

system	20 000 partitions				765 partitions			
value	*λ*		copies		*λ*		copies	
repeats	lower	upper	lower	upper	lower	upper	lower	upper
1	0,0030	8,86	61	177297	0,077	5,51	59	4218
2	0,0014	9,51	29	190270	0,038	5,31	29	4063
3	0,0012	9,92	23	198378	0,030	5,31	23	4063
4	0,0011	10,00	21	200000	0,020	6,53	15	4993

The table lists the lowest and highest values (as *λ* and as number of target copies per reaction) at which 95% of all reactions contain less than 25% error. The values were calculated for a system with 20 000 partitions and for a system with 765 partitions. A conversion of these numbers into ng DNA per reaction for some common crops in given in S4 Table.

### Fraction of sample compartmentalized

Both droplet and chamber based dPCR systems are subject to variability in the number of compartments that are generated and/or accepted into the analysis. On top of that, most compartmentalization techniques have a certain ‘dead volume’. As a consequence, and unlike in real time PCR, not the entire volume of sample loaded into the chamber/droplet generator is analysed. This essentially corresponds to a form of sub-sampling which may in turn add variation or error the quantification, especially for reactions with targets at very low abundance. We can express the number of compartments in the analysis in a relative way, *i.e.* as the fraction of sample compartmentalized: the total volume of droplets accepted into the analysis divided by the total volume loaded into the device.

**Fig 8:**
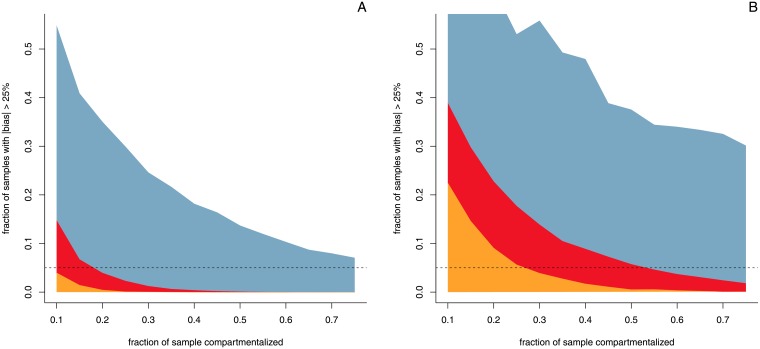
Probability of error in function of sample compartmentalisation. The variability in positive and negative droplets when only part of the samples is analysed may cause error in the quantification result. The figures show the fraction of results that have more than 25% error (10 000 parametric bootstraps per data point) for different amounts of compartmentalisation and for different percentages of analyte. The yellow area represents 1% of analyte, red 0.5%, and blue 0.1%. The horizontal line represents the 5% criterion. **Panel A** shows the results for endogene *λ* = 3 (target *λ* = 0.03, 0.015, and 0.003) **Panel B** shows the results for endogene *λ* = 1 (target *λ* = 0.01, 0.005, and 0.001).

To inspect whether this within-run sub-sampling has any significant effect on the quantification of a target, we simulated sub-sampling for various percentages of analyte and various values of endogene *λ*. For the purpose of the simulation we considered a digital PCR with the specifications of the Biorad QX200 platform: *in silico* we generate the maximum number of droplets from a Poisson distribution (*i.e.* 23530 0.85 nl droplets or 20*μ*l total) from which we randomly sub-sampled a certain fraction. The latter droplets are then used for target quantification. For each fraction of compartmentalization inspected, 10000 of such parametric bootstraps were performed, which were then inspected to see how many had more than 25% error (see Fig 8).

Unsurprisingly, results show that there is an increase in variability as less of the total sample is compartmentalized. However, for *λ* = 3 and 1% analyte, we have to go down to very low levels of compartmentalization (< 10%) before the chance of excessive error (> 25%) becomes greater than 1 in 20 (*i.e.* 95% of the simulations have less than 25% relative error). For lower levels of analyte, more compartmentalization is required to keep 95% of the simulations below 25% error, and the same is true for lower values of *λ*.

From the results presented in Fig 8) we propose to require at least 30% compartmentalisation (≈7000 droplets) for quantification down to 1%, and 50% compartmentalisation for quantification down to 0.5% (≈11800 droplets).

### On the usefulness of Poisson confidence intervals

The confidence bounds as calculated by dPCR software are based on the variability of the Poisson process alone: they only take into account the variability of the (random) sampling processes that distribute the targets over the partitions. The Poisson statistics also allow to ‘merge’ reactions to improve quantification (*i.e.* assume *λ* is identical across several reactions and sum up their positive and negative partitions). The latter is a useful aspect of digital PCR, especially when large volumes of sample have to be analysed to generate sufficient positive partitions. However, merging can also be used at high values of *λ* to yield more precise estimates with very narrow confidence bounds. In [16] the authors point out that this theoretical model does not account for all sources of variation and thus may underestimate the variance and overestimate the coverage of the confidence intervals. Indeed, since the CI are based on the variability of the Poisson process alone, they do not take into account the various other sources of variability that are present in the analytical chain.

Starting from a DNA sample at stock concentration the analytical chain includes at least (I) the dilution to a suitable concentration, (II) the preparation of the reaction mix, and (III) the distribution of the mix across the partitions. Each of these three steps may represent the introduction of additional variation into the process which will ultimately lead to a higher dispersion in the final estimates of the amount of target copies.

In order to gauge the possible impact of the additional variation on the coverage of the Poisson confidence intervals we prepared 48 independent dilutions of the same soy sample which contained two targets in a 1:50 ratio (GTS 40-3-2 and the soy endogene *le1*). Each dilution was analysed in 4 repeats using a duplex reaction. The original DNA stock was also analysed in 48 independent. This set-up allows us to compare the coverage of the Poisson CI at (I) different levels of target concentration, (II) at different levels of merging.

**Fig 9:**
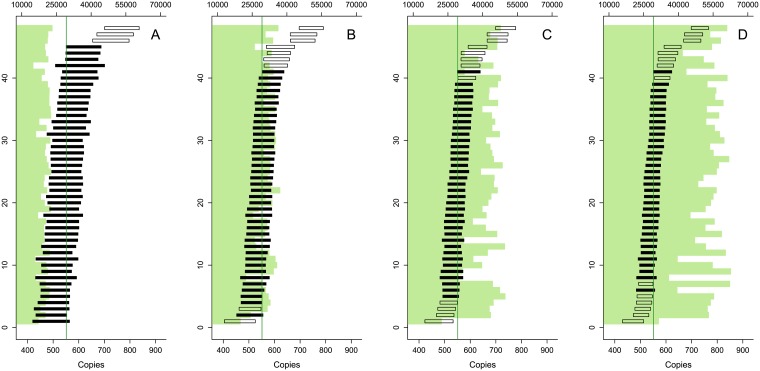
Poisson Confidence intervals for 48 independent dilutions of a rare target. The bottom axis shows the number of copies in the reaction, the top axis shows the number of droplets analysed (as indicated by the green bar-plots). The vertical line indicates the median estimate, confidence intervals that contain this value are coloured black, confidence intervals that do not contain this value are represented as empty boxes. **Panels A through D** show the decrease in confidence width as a consequence of merging the reactions, the respective CI coverage factors for the panels are: 93.75, 81.25, 75, and 70.83 percent.

**Fig 10:**
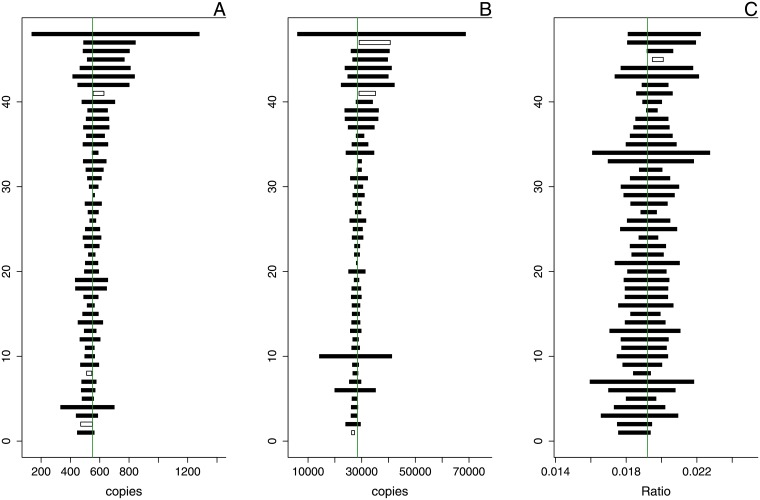
Confidence intervals for 48 independent dilutions based on standard deviation of repeated measurement. The vertical line indicates the median estimate, confidence intervals that contain this value are coloured black, confidence intervals that do not contain this value are represented as empty boxes. **Panel A** shows the results for a rare target, **Panel B** shows the results for an abundant target, and **Panel C** show the results of their ratio, the respective CI coverage factors for the panels are: 93.75, 93.75, and 97.92 percent.

Results of this experiment are presented in Figs 9 and 10, and S6 and S7 Figs.

The total variance in digital PCR results as observed from these repeated reactions ($\sigma_{total}^{2}$) may be seen as consisting of two major components: the variance due to the random distribution of targets over the partitions ($\sigma_{Pois}^{2}$) and the variance due to dilution and preparation ($\sigma_{Prep}^{2}$): $\sigma_{total}^{2}\approx \sigma_{Pois}^{2}+\sigma_{Prep}^{2}$. Our results indicate that at low levels of analyte, the Poisson variation ($\sigma_{Pois}^{2}$) is the dominant process and experimental variation ($\sigma_{Prep}^{2}$) only contributes marginally to the total variation of the estimates. As a consequence, the coverage of the confidence intervals for the estimated number of target copies based solely on Poisson variation is close to the intended 95% (93.75%, see Fig 9A). However, at high levels of analyte, experimental variation dominates over Poisson variation and as a consequence, coverage of the confidence intervals is far below expected (25%, see S6A Fig). In both cases merging replicates decreases the CI width and in most cases this will further decrease the coverage of the Poisson CI (see Fig 9A through Fig 9D). Merging reactions therefore seems a valid option in the detection of very rare targets (*e.g.* when very large amounts of sample have to be analysed to generate sufficient positive partitions), but may lead to overestimating the confidence in one’s results when quantifying more abundant targets.

In contrast to the confidence intervals for the absolute copy estimates, the ratio confidence intervals based upon the former *do* provide the intended coverage (100%, see S7 Fig), even when merging all repeats (97.92%). The cause for this is the use of Fieller’s theorem to calculate the ratio confidence intervals: the widest confidence interval of the two values involved contributes most to the width of the ratio CI. In our case, the *λ* with the widest confidence interval is that of the transgene. For the latter the coverage was close to its expected value, which explains why this is also the case for the coverage of the ratio CI.

### An improved strategy for dPCR confidence intervals

Rather than rely on the Poisson statistics it is more useful to rely on repeated analysis to gauge the variability of the estimates, especially at higher values of *λ*. Current sample preparation practice in the field of food analysis involves the original sample be split in two sub-samples (*A* and *B*) which are both extracted for DNA. Both extracts are then analysed by real time PCR in duplicate, yielding results (*A*_1_, *A*_2_) and (*B*_1_, *B*_2_). This practice captures the most important levels of variability in a very economic way.

When applying the same strategy to dPCR, this yields 4 quantifications that reflect both sub-sampling and pipetting variability. Calculating the mean ($\hat{\mu }$) and standard deviation ($\hat{\sigma }$) allows for the construction of somewhat ‘ad hoc’ confidence intervals ($\hat{\mu }\pm 1.96\cdot \hat{\sigma }$). However, these confidence intervals better capture the variability present in the analytical chain than the Poisson confidence intervals (see Fig 10). This approach works for both single target and ratio quantification and should be preferred over relying on Poisson variation alone when evaluating quantification results.

## Conclusion

Digital PCR is rapidly becoming a routine tool in DNA based food and feed analysis. Harmonization has always been one of the trump cards of European food safety. Therefore, similar to qPCR, we aimed to set guidelines and performance parameters for the acceptance and validation of analytical methods in this field.

We have inspected several critical aspects of the digital PCR data output and evaluated which criteria should be met to ensure reliable quantification and data quality evaluation on a run-by-run basis. Based on the concepts of misclassification and variability we recommend that digital PCR assays are validated at a *λ* value of 0.7. In the resulting data **(I)** there should only be two fluorescence populations, **(II)** the peak resolution should be at least 2.5, and **(III)** less than 2.5% of the total number of droplets generated should classify as ‘rain’.

Our results further indicate that the one of the mechanisms leading to excessive amounts of ‘rain’ may be sequence dependent (either target or primer sequence). As a consequence, re-designing primers for a certain target may be the most effective way in obtaining a robust dPCR assay. Should the choice of target sequence be limited, sonication and/or extended cycling regimes may help in improving an assay.

In addition, we found that compartments with intermediate fluorescence (rain) are likely to contain the target sequence and should be classified as positive. As a consequence of the varying kurtosis observed in the distribution of the fluorescence values, it does not seem possible to give a single answer in terms of how many *σ* above the median negative fluorescence value the thresholds should be placed. Instead, we can only recommend to set the threshold as close as reasonably possible to the negative cloud.

In post run analysis, we recommend to inspect **(I)** the concentration of the target analyte and **(II)** the degree of compartmentalization in order to have an approximate 95% chance to have less than 25% relative error on the final quantification result. Specifically, there should be between about 30 and 19000 (4000) targets for a 20 000 (765) compartment system (assuming analyis with 2 repeats) and there should be at least 30% compartmentalisation for quantification down to 1%, and 50% compartmentalisation for quantification down to 0.5%.

Finally, we have shown that the Poisson confidence bounds for droplet digital underestimate the total amount of variability in the analysis. A more apt strategy to digital PCR confidence intervals involves the original sample be split in two sub-samples which are both extracted for DNA. Both extracts are subsequently analysed in duplicate, and the standard deviation across all four results is used to obtain confidence bounds around the mean.

These guidelines put numbers on what many digital PCR users already know and practise. It gives them further tools to define and choose the best methods for their work. The authors hope that the whole of these recommendations may help flawless integration of dPCR in routine analysis.

## Supporting Information

S1 Table Primer pairs used in this study. F indicates the forward primer, R the reverse, and P the probe. For the latter, the transgenes used FAM-TAMRA labelling, except for GTS 40-3-2 (FAM-MGB) and *le1* (HEX-BHQ). ‘Target’ specifies the intended DNA target. ‘[conc]’ is the final concentration (in nM) of the primer/probe in the reaction mix. ‘Length’ denotes the length of the amplicon in basepairs. ‘Ref’ indicates from which publication the respective primers were taken. The soybean endogene *le1* (lectin) encodes a prevalent seed protein. The maize endogene *hmg* (high mobility group) encodes a chromatin-associated protein. The oilseed rape endogene *cruA* (cruciferin) encodes a seed storage protein. The cotton endogne *acp1* (acyl carrier protein) encodes a component associated with fatty acid biosynthesis. The event specific methods target a fragment covering the junction between the insert and the plant genome.
(TEX)

S2 Table Results for dPCR primer concentration optimization. Four primer concentrations were tested (150, 300, 450, and 600 nM) in duplicate. Probe concentrations are mentioned per target, as are resolution, rain to total droplet ratio, and the number of droplet populations as determined by the algorithm. Probe concentrations are such that the primer to probe ratio is the same as for the validated conditions (see S1 Table).
(TEX)

S3 Table Results for dPCR primer annealing/extension temperature optimization. Eight technical repeats of each method were run at different temperatures each (62, 61.6, 60.9, 59.8, 58.4, 57.3, 56.5, and 56°C). Reaction parameters shown are: digital resolution, rain to total droplet ratio, and the number of droplet populations as determined by the algorithm. Primer concentration was 450 nM for all reactions, probe concentrations are such that the primer to probe ratio is the same as for the validated conditions (see S1 Table).
(TEX)

S4 Table DNA load per reaction for two digital PCR systems (20 000 and 765 compartments) for seven common crops. The table shows the minimum, optimal (*λ* = 1.59), and maximal number of nanograms per crop as calculated from Table 2, using 1C values from [3, 25].
(TEX)

S1 Fig Effect of dilution on the amount of rain. Six steps of two-fold dilution (*λ* 0.7 to about 0.02), droplet readout (Fluorescence Units) is shown. Each colour represents a subsequent dilution step. **Panel A** shows the results for TC1507. **Panel B** shows the results for Mon88017.
(EPS)

S2 Fig Running additional cycles in digital PCR. Each panel shows the observed droplets for 4 cycling regimes: 45, 60, 75, and 90 cycles. All reactions were run at *λ* ≈ 0.7. **Panel A** shows the results for TC1507. **Panel B** shows the results for MON880107.
(EPS)

S3 Fig Error as a result of droplet misclassification (positives misclassified as negative). The effect of 1% misclassification on a 20 000 partition system is shown for absolute quantification at various levels of lambda. The error introduced is minimal around *λ* ≈ 1, for higher values of lambda the error increases mostly due to decreasing true negatives, whereas for lower values of lambda the error increases mostly due to decreasing true positives.
(EPS)

S4 Fig Effect of sonication on the amount of rain. Six conditions of increasing sonication were tested (ranging from 0 seconds to 15 seconds in steps of 3 seconds), droplet readout (Fluorescence Units) is shown. Each colour represents a subsequent dilution step. **Panel A** shows the results for TC1507. **Panel B** shows the results for MON88017.
(EPS)

S5 Fig Effect of PCR enhancers on ddPCR droplet readout. Each colour represents a different condition: from left to right: nothing, 2% DMSO, 0.2M Trehalose. **Panel A** shows the results for TC1507. **Panel B** shows the results for MON88017.
(EPS)

S6 Fig Poisson Confidence intervals for 48 independent dilutions of an abundant target. The bottom axis shows the number of copies in the reaction, the top axis shows the number of droplets analysed (as indicated by the green barplots). The vertical line indicates the median estimate, confidence intervals that contain this value are marked black, confidence intervals that do not contain this value are represented as empty boxes. **Panels A through D** show the decrease in confidence width as a consequence of merging the reactions, the respective CI coverage factors for the panels are: 25, 25, 29.17, and 16.67 percent.
(EPS)

S7 Fig Ratio confidence intervals for 48 independent dilutions based on Poisson confidence intervals. The bottom axis shows the endogene to transgene ratio, the top axis shows the number of droplets analysed (as indicated by the green barplots). The vertical line indicates the median estimate, confidence intervals that contain this value are marked black, confidence intervals that do not contain this value are represented as empty boxes. **Panels A through D** show the decrease in confidence width as a consequence of merging the reactions, the respective CI coverage factors for the panels are: 100, 100, 100, and 97.92 percent.
(EPS)

S1 File S1_File.pdf. PDF file containing the CTAB based DNA extraction protocol that was used for the preparation of samples in this publication.
(PDF)

S2 File S2_File.R. ‘S2_File.R’ contains a short function that can be used to read the Bio-rad Quantasoft output (as exported via ‘options > export amplitude and cluster data’) into R. Within the file, commented text provides annotations and instructions for the use of this algorithm. In addition, there is a github page (http://www.github.com/Gromgorgel/ddPCR) that contains further instructions, R-code examples, as well as the entire data set underlying the findings this publication.
(R) Click here for additional data file.

S3 File S3_File.R. ‘S3_File.R’ contains the main droplet categorization and quantification function that was used to evaluate the digital PCR reactions. Within the file, commented text provides annotations and instructions for the use of this algorithm. In addition, there is a github page (http://www.github.com/Gromgorgel/ddPCR) that contains further instructions, R-code examples, as well as the entire data set underlying the findings this publication.
(R) Click here for additional data file.

S4 File S4_File.R. ‘S4_File.R’ contains the algorithms for the simulations and calculations that were mentioned throughout the text of the main article. Within the file, commented text provides annotations.
(R) Click here for additional data file.

S5 File S5_File.pdf. PDF file containing the definitions of the discarded performance criteria and the instructions for the manual calculation of the resolution of a digital PCR reaction.
(PDF)
