# Supplementary material for: Measuring Digital PCR Quality: Performance Parameters and Their Optimization
Source: PLoS One. 2016 May 5;11(5):e0153317. doi: 10.1371/journal.pone.0153317 (PMC4858304; doi:10.1371/journal.pone.0153317)
Supplement: S1 File — PDF file containing the CTAB based DNA extraction protocol that was used for the preparation of samples in this publication. (PDF) [file pone.0153317.s012.pdf]

MEASURING DIGITAL PCR QUALITY: PERFORMANCE PARAMETERS AND THEIR OPTIMIZATION SUPPLEMENTAL MATERIAL FILE S1

Lievens, A. *[1], Jacchia, S.[1], Kagkli, D.[1], Savini, C.[1], and Querci, M.[1]

[1]Molecular Biology and Genomics Unit, European Commission - Joint Research Centre, Institute for Health and Consumer Protection

CTAB DNA extraction protocol

The extraction protocol given on the following pages is based on the one published in [1] and optimised for extraction of DNA from dry, powder-like, materials (*e.g.* certified reference materials), especially maize and soy flours. The volumes given take into account the absorption of buffer by the material. However, should the plant material absorbs so much of the buffer that the volumes mentioned in the protocol cannot be recovered after centrifugation, 1 to 3 ml of water may be added to the powder prior to extraction.

Buffers

Extraction Buffer (final volume: 1 liter)

CTAB:	20 g/l (2%)
NaCl (5 M)	280 ml (1,4 M final)
Tris (1M, pH 8)	100 ml (0,1 M final)
EDTA (0.5M, pH 8)	40 ml (0,02 M final)
ddH2O	to 900 ml
adjust pH to 8	NaCl and HCl
ddH2O	to 1000 ml

Precipetation buffer (final volume: 1 liter)

CTAB:	5 g/l (0.5%)
NaCl (5 M)	8 ml (0.04 M final)
ddH2O	to 1000 ml

Other reagents

Proteinase K	20 mg/ml
RNAse A	20 mg/ml
Isopropanol	100%
EtOH	70%
NaCl Solution:	70,1 g/l (1,2M)
Chloroform:isoamyl alcohol	24:1

Material needed

tubes	50 ml
aliquots	2 or 5 ml
centrifuge	
heating block	
Speedvac	

All equipment should be able to accommodate 50 ml tubes (except for the speedvac).

*Corresponding author. Tel.: +39 0332 78 3641
E-mail: antoon.lievens@ec.europa.eu

Extraction Protocol

Use 50 ml tubes resistant to high speed centrifugation.
Amount of starting material: 3 g

Add 16 ml extraction buffer.
Add 80 μl Proteinase K (100 μg/ml final) vortex & incubate at 65°C for 50min.
Add 80 μl RNAse A (100 μg/ml final) vortex & incubate at 65°C for 10min.

Centrifuge 15 minutes at $\geq$ 10 000 $\times$ g.
Transfer a minimum of 8 ml supernatant to a new tube.

Add 4 ml chloroform:isoamyl (24:1) and vortex.
Centrifuge 10 minutes at $\geq$ 5 000 $\times$ g.
Transfer 7 ml supernatant to a new 50 ml tube.

Add 14 ml (2 volumes) precipitation buffer, invert 3-4 times.
Incubate 1h at room temperature.
Centrifuge 15 minutes at $\geq$ 10 000 $\times$ g.
Remove supernatant.

Add 3 ml NaCl Solution to redissolve DNA.
(Incubate 30 minutes at 50°C with regular shaking for a more complete re-suspension)
Add 3 ml chloroform:isoamyl (24:1), vortex.
Centrifuge 10 minutes at $\geq$ 5 000 $\times$ g.
Transfer 2.5 ml supernatant to a new 5 ml eppendorf tube (or 2$\times$ 1.25ml to two 2 ml eppendorf tubes).

Add 1.5 ml (0,6 volumes) isopropanol (or 750 μl to each 2 ml tube).
Incubate 10 minutes at room temperature.
Centrifuge 15 minutes at $\geq$ 10 000 $\times$ g.
Remove supernatant.

Wash pellet with 2 ml 70% EtOH (or 1ml per 2ml tube) for 10 min to overnight (e.g. in a tube rotator).
Centrifuge 10 minutes at $\geq$ 10 000 at $\geq$ g.
Remove supernatant.

Vacuum dry pellet 10 minutes at 30°C.
Resuspend in 500 μl (or 250 μl per 2ml tube) 0.1$\times$ TE.

References

1. J. Sambrook and D. W. Russel. *Molecular Cloning: A Laboratory Manual 3rd Ed.* Cold Spring Harbor Laboratory Press., Cold Spring Harbor, NY, 2001.
