# Supplementary material for: Measuring Digital PCR Quality: Performance Parameters and Their Optimization
Source: PLoS One. 2016 May 5;11(5):e0153317. doi: 10.1371/journal.pone.0153317 (PMC4858304; doi:10.1371/journal.pone.0153317)
Supplement: S5 File — PDF file containing the definitions of the discarded performance criteria and the instructions for the manual calculation of the resolution of a digital PCR reaction. (PDF) [file pone.0153317.s016.pdf]

Measuring digital PCR quality: Performance Parameters and their Optimization Supplemental material File S5

Lievens, A. *[1], Jacchia, S.[1], Kagkli, D.[1], Savini, C.[1], and Querci, M.[1]

[1]Molecular Biology and Genomics Unit, European Commission - Joint Research Centre, Institute for Health and Consumer Protection

Discarded performance criteria

Gain is a concept from electronics which captures the ability of an amplifier to increase the amplitude of a signal. It translates well to PCR where the intensity of the fluorescence is amplified. Although it provides information on the amount of amplification of the fluorescence signal, it does not give a measure of how well the populations are separated. This parameter was abandoned in favour of the peak resolution. The definition of gain relevant to dPCR is:

$$gain = 10 \cdot \log \frac{\bar{FU}_p}{\bar{FU}_n} \tag{1}$$

where $\bar{FU}_p$ is the average fluorescence of the positive population and $\bar{FU}_n$ is the average fluorescence of the negative population.

The signal to noise ratio (SNR) is a concept from signal processing and engineering which compares the level of desired signal to noise. It is more difficult to apply to dPCR since the negative droplets are not noise but are informative as well. One useful definition is

$$SNR_a = \frac{\mu_a}{\sigma_a^2} \tag{2}$$

which can be used to calculate separate SNR for both populations, giving a measure of the dispersion of the fluorescence values within each group. Again, the latter does not really inform the user if the two populations are well separated or not.

*Corresponding author. Tel.: +39 0332 78 3641
E-mail: antoon.lievens@ec.europa.eu

Manual calculation of digital resolution

By moving the threshold, the user can determine: the lowest fluorescence measurement ($F_{N,0}$) and the highest fluorescence measurement ($F_{P,1}$). In the same way, the highest fluorescence of the negative cloud ($F_{N,1}$) and the lowest fluorescence of the positive cloud ($F_{P,0}$) have to be determined, note that for these two points we ignore the 'rain' (see figure 1). The relative 'empty' bandwidth is then given by:

$$Bw_0 = \frac{F_{P,0} - F_{N,1}}{F_{P,1} - F_{N,0}} \tag{3}$$

From which the resolution can be calculated as:

$$R_s = -\frac{Bw_0 + 1}{Bw_0 - 1} \tag{4}$$

This way only four data points have to be collected per run. Since we define the 'rain' as the population of the empty band (*i.e.* droplets whose fluorescence measures between $F_{N,1}$ and $F_{P,0}$), these fluorescence measurements will also be used to obtain the second performance criterion (percentage rain).

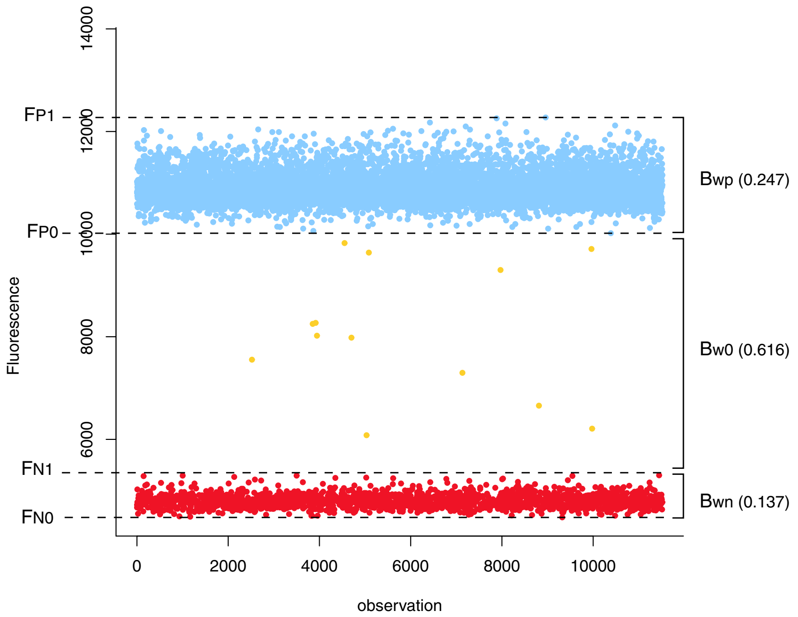

Figure 1: Illustration of the manual determination of dPCR resolution. The lower population of droplets is considered negative (red, between limits $F_{N,1}$ and $F_{N,0}$). The higher population is considered positive (blue, between limits $F_{P,1}$ and $F_{P,0}$). The droplets with fluorescence values between $F_{N,1}$ and $F_{P,0}$ are considered 'rain' (orange).
